# PRMT4-mediated arginine methylation promotes tyrosine phosphorylation of VEGFR-2 and regulates filopodia protrusions

**DOI:** 10.1016/j.isci.2022.104736

**Published:** 2022-07-11

**Authors:** Edward Hartsough, Rajani R.J. Shelke, Razie Amraei, Zahra Aryan, Saran Lotfollahzadeh, Nader Rahimi

**Affiliations:** 1 Department of Pathology and Laboratory Medicine, Boston University Medical Campus, Boston, MA 02118, USA; 2 Renal Section, Department of Medicine, Boston University Medical Center, Boston, MA 02118, USA

**Keywords:** Biochemistry, Molecular physiology, Cell biology

## Abstract

Through tightly controlled multilayer mechanisms, vascular endothelial growth factor receptor-2 (VEGFR-2) activation and its downstream signal transduction govern vasculogenesis and pathological angiogenesis, such as tumor angiogenesis. Therefore, it is critical to understand the molecular mechanisms governing VEGFR-2 signal transduction. We report that protein arginine methyltransferase 4 (PRMT4) via its highly conserved EVH1 and PH domain-like N-terminal domain binds to VEGFR-2 and mediates methylation of the juxtamembrane arginine 817 (R817) on VEGFR-2. Methylation of R817 selectively increases phosphorylation of tyrosine 820 (Y820). Phosphorylation of Y820 facilitates the c-Src binding with VEGFR-2 via Src homology domain 2 (SH2). Interfering with the methylation of R817 or phosphorylation of Y820 inhibits VEGFR-2-induced filopodia protrusions, a process that is critical for the core angiogenic responses of VEGFR-2. Methylation of R817 is an important previously unrecognized mechanism of the angiogenic signaling of VEGFR-2, with implications for the development of novel-targeted VEGFR-2 inhibitors.

## Highlights

• Arginine 817 methylation regulates phosphorylation of Y820 on VEGFR-2
• Phosphorylation of Y820 recruits c-Src kinase to VEGFR-2, leading to its activation
• VEGFR-2/c-Src axis mediates filopodia protrusions in endothelial cells

## Graphical abstract

**Figure:**
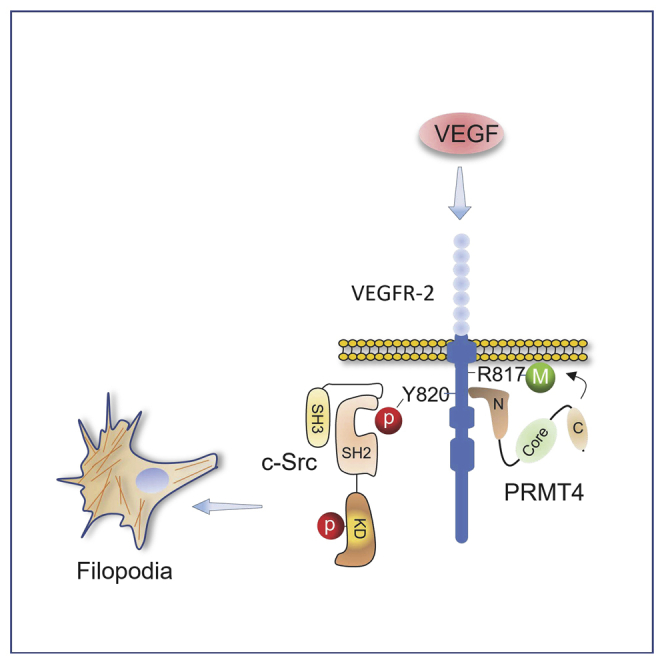

## Introduction

Vascular endothelial growth factor receptor-2 (VEGFR-2) is a key receptor tyrosine kinase (RTK) that plays a major role in vasculogenesis and angiogenesis. It stimulates various cellular responses in endothelial cells, including cell proliferation, survival, migration, and differentiation (Cleaver and Melton, 2003; Conway et al., 2001). VEGFR-2 undergoes extensive posttranslational modifications (PTMs) including phosphorylation, methylation, acetylation, and ubiquitination (Koch et al., 2011; Rahimi and Costello, 2015). VEGFR-2 is heavily phosphorylated at tyrosine and serine sites; however, the molecular regulation of these sites, except few, remains largely unknown. While activation loop tyrosine autophosphorylation sites, Tyr1054 and Tyr1057, are required for the kinase activation of VEGFR-2 (Meyer et al., 2008), phosphorylation of Tyr1175 recruits multiple Src homology domain (SH2)-containing proteins such as phospholipase-Cγ (PLCγ1) (Meyer et al., 2003; Takahashi et al., 2001), SHC, GRB2 (Warner et al., 2000), Shb adaptor protein (Holmqvist et al., 2004), and the p85 subunit of PI3 kinase (Dayanir et al., 2001) to VEGFR-2. Moreover, VEGFR-2 is phosphorylated on several serine/threonine sites (Rahimi and Costello, 2015), and phosphorylation of two distinct serine residues, Ser1183 and Ser1189, recruits βTRCP ubiquitin E3 ligase complex and promotes degradation of VEGFR-2 (Meyer et al., 2011).

Our previous study identified five methylation sites on VEGFR-2, including Arg817, Lys856, Lys861, Lys1041, and Arg1115 (Hartsough et al., 2013). Lys1041 is located in the kinase domain of VEGFR-2, and its methylation regulates the kinase activation of VEGFR-2 (Hartsough et al., 2013). The roles of the remaining methylated residues, in particular, Arg methylation, on the VEGFR-2 function remain unknown. Methylation of Arg and Lys is catalyzed by protein arginine methyltransferases (PRMTs) and lysine methyltransferases (KMTs), respectively. Previous studies have shown that PRMTs play key roles in the regulation of angiogenesis; endothelial-specific loss of PRMT1 in mice causes angiodysplasia (Ishimaru et al., 2017), PRMT4 activity is linked to upregulation of VEGF (Yan et al., 2021), and inhibition of PRMTs via pharmacological agents blocks angiogenesis (Balcerczyk et al., 2016).

In this study, we have focused on the methylation of Arg817 of VEGFR-2 and demonstrate that PRMT4 catalyzes methylation of VEGFR-2 at Arg817. Methylation of Arg817 modulates phosphorylation of Tyr820, increases the recruitment of Src kinase to VEGFR-2, leading to c-Src activation. Interfering with the methylation of R817 inhibited VEGFR-2-induced filopodia protrusions, a key cellular event that is required for VEGFR-2-induced angiogenic signaling.

## Results

### PRMT4 interacts with VEGFR-2

**Figure 1:**
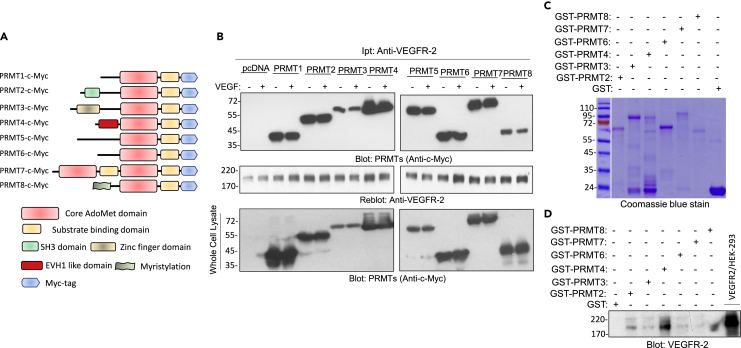
PRMT4 interacts with VEGFR-2 (A) Schematic Myc-tagged PRMT constructs. (B) HEK-293 cells expressing VEGFR-2 were transfected with C-terminus Myc-tagged PRMT constructs as shown. After 48 h, cells were lysed and whole cell lysates (WCL) were immunoprecipitated with anti-VEGFR-2 antibody and blotted with anti-*c*-Myc antibody for PRMTs. The same membrane was re-blotted for VEGFR-2. WCL from the same experiment was blotted for PRMTs via anti-*c*-Myc antibody. (C) Expression of purified recombinant GST-PRMT proteins in *E. coli*. (D) Whole cell lysate from HEK-293 cells expressing VEGFR-2 was subjected to GST-pulldown assay using GST alone or GST-PRMTs as indicated, followed by Western blot analysis using anti-VEGFR-2 antibody.

Our previous LC-MS/MS analysis has identified five methylated residues on VEGFR-2, including two arginine sites (R817 and R1115) and three lysine sites (K856, K861, and K1041) (Hartsough et al., 2013). Methylation of K1041 modulates tyrosine autophosphorylation sites (Y1052 and Y1054) located in the activation loop (Hartsough et al., 2013). However, the role of other methylation residues, in particular, arginine methylation sites on VEGFR-2 and a particular protein arginine methyltransferase involved in the methylation of VEGFR-2 remains unknown. To identify a particular PRMT involved in the arginine methylation of VEGFR-2, we generated HEK-293 cell lines-expressed VEGFR-2 alone or co-expressed VEGFR-2 with one of the C-terminus c-Myc-tagged PRMTs (Figure 1A). To address the binding of PRMTs with VEGFR-2, the cells were lysed, and the whole cell lysates were subjected to an immunoprecipitation assay via an anti-VEGFR-2 antibody. After extensive washing (4X) with the lysis buffer containing 1% sodium dodecyl sulfate (SDS), the protein-A Sepharose-bound proteins were resolved on SDS-PAGE, followed by immunoblotting with an anti-*c*-Myc antibody. The result showed that all the PRMTs (1–8) were co-immunoprecipitated with VEGFR-2 with or without VEGF stimulation (Figure 1B), indicating that they interact with VEGFR-2 in a constitutive manner. Whole cell lysates from the same cell lines were also blotted for the expression of PRMTs (Figure 1B).

The observation that all the PRMTs were co-immunoprecipitated with VEGFR-2 suggested to us that PRMTs either share a common motif/domain that enables their interaction with VEGFR-2 or the observed interaction of PRMTs with VEGFR-2 occurs in a non-specific manner. To address the specificity of the binding of PRMTs with VEGFR-2, we generated a panel of GST-fusion recombinant PRMTs (Figure 1C) and tested for their ability to interact with VEGFR-2 via GST-pulldown assay. The result showed that although PRMT2, PRMT3, PRMT6, PRMT 7, and PRMT8 were weakly interacted with VEGFR-2, the strongest interaction was observed between PRMT4 and VEGFR-2 (Figure 1D). Taken together, these observations indicate that PRMPT4 interacts with VEGFR-2 in cell culture (co-immunoprecipitation assay) and *in vitro* GST-pulldown assay. Other PRMTs could also interact with VEGFR-2, albeit weakly.

### PRMT4 N-terminus domain selectively mediates its binding with VEGFR-2

**Figure 2:**
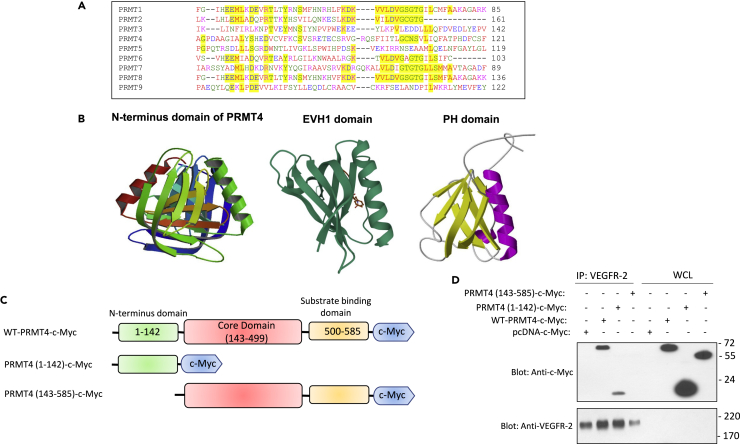
The N-terminus domain of PRMT4 mediates the interaction of PRMT4 with VEGFR-2 (A) The N-terminal amino acid sequence alignment of human PRMTs. (B) The N-terminus crystal structure of PRMT4, EVH1 domain, and PH domain. (C) Schematic of wild-type and truncated PRMT4 constructs. (D) HEK-293 cells expressing VEGFR-2 were transfected with PRMT4 constructs as shown. After 48 h post-transfection, cells were lysed and subjected to co-immunoprecipitation assay via anti-VEGFR-2 antibody followed by Western blot analysis using a c-Myc antibody. The same membrane was re-blotted with anti-VEGFR-2 antibody. Whole cell lysate (WCL).

**Figure 3:**
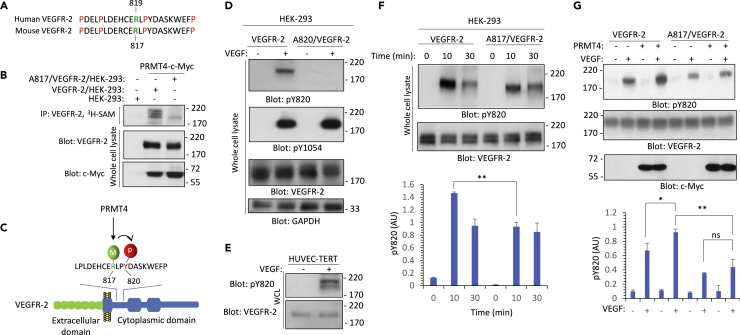
Methylation of arginine 817 modulates phosphorylation of VEGFR-2 on tyrosine 820 (A) Arginine 817 (R817) is conserved on human and mouse VEGFR-2. (B) HEK-293 cells expressing VEGFR-2 or Arg 817 mutant VEGFR-2 (A817/VEGFR-2) were lysed, and VEGFR-2 purified via immunoprecipitation with anti-VEGFR-2 antibody. The purified VEGFR-2 proteins were subjected to an *in vitro* methylation assay, plus semi-purified PRMT4 (purified via immunoprecipitation). The whole cell lysates from the same experimental group were blotted for VEGFR-2 and PRMT4, respectively. (C) Schematic of the proposed model for PRMT4-mediated methylation of VEGFR-2 and its role in the regulation of tyrosine 820 (Y820) phosphorylation. (D) Serum-starved HEK-293 cells expressing wild-type VEGFR-2 or tyrosine mutant VEGFR-2 (A820/VEGFR-2) were stimulated with VEGF for 10 min, cells were lysed, and whole cell lysates were subjected to Western blot analysis using anti-phospho-Y820, anti-phospho-Y1054, anti-VEGFR-2, or GAPDH antibodies. (E) Serum-starved HUVEC-TERT cells were stimulated with VEGF for 10 min, cells were lysed, and whole cell lysates were subjected to Western blot analysis using anti-phospho-Y820 or anti-VEGFR-2 antibodies. (F) Serum-starved HEK-293 cells expressing wild-type VEGFR-2 or arginine mutant 817 VEGFR-2 (A817/VEGFR-2) were stimulated with VEGF for 0, 10, and 30 min, cells were lysed, and whole cell lysates were subjected to Western blot analysis using anti-phospho-Y820 or anti-VEGFR-2 antibodies. The graph is representative of three independent experiments. (G) Serum-starved HEK-293 cells expressing wild-type VEGFR-2 or arginine 817 mutant VEGFR-2 (A817/VEGFR-2) alone or co-expressed with PRMT4-*c*-Myc were stimulated with VEGF for 10 min, cells were lysed, and whole cell lysates were subjected to Western blot analysis using anti-phospho-Y820, anti-phospho-Y1054, anti-VEGFR-2, or anti-*c*-My antibodies. The graph is representative of three independent experiments. Not significant (ns). ∗p-value less than 0.05; ∗∗p-value less than 0.01; ∗∗∗p-value less than 0.001.

To identify the potential region on PRMTs involved in the interaction with VEGFR-2, we aligned the amino acid sequence of PRMTs. There was a significant degree of homology in the N-terminus of PRMTs (Figure 2A). However, the N-terminal of PRMT4 was more divergent compared to other PRMTs (Figure 2A). Furthermore, the crystal structure of PRMT4 has been previously reported to contain a pleckstrin homology (PH, two perpendicular antiparallel β-sheets followed by a C-terminal helix)-like domain, which is also similar to the drosophila-enabled/vasodilator-stimulated phosphoprotein homology 1 (EVH1) domain (Figure 2B). While the PH domain is known to interact with the phospholipids (Singh et al., 2021), the EVH1 domain interacts with the proline-rich motifs (Ball et al., 2002; Peterson and Volkman, 2009). Interestingly, most of the PRMT4 substrates also contain proline-rich motifs (Shishkova et al., 2017). These observations suggest that PRMT4 via its N-terminus domain can recognize VEGFR-2 and catalyze methylation of VEGFR-2 at R817, which is also surrounded by the proline residues (Figure 3A).

To examine the hypothesis that the N-terminal of PRMT4 mediates the binding of PRMT4 with VEGFR-2, we generated two truncated PRMT4 constructs encompassing the N-terminal alone (PRMT4-1-142) or PRMT4 without the N-terminus (PRMT4-143-585) (Figure 2C). We co-expressed these constructs with VEGFR-2 and analyzed their interactions with VEGFR-2 via a co-immunoprecipitation assay. The result showed that N-terminal PRMT4 alone is sufficient to interact with VEGFR-2 (Figure 2D). Deletion of the N-terminal abolished the interaction of PRMT4 with VEGFR-2 (Figure 2D), demonstrating that PRMT4 via its PH/EVH1-like N-terminus domain interacts with VEGFR-2.

### PRMT4 mediates methylation of R817 on VEGFR-2

Next, we tested the hypothesis that the association of PRMT4 with VEGFR-2 could mediate methylation of R817, which is conserved both in human and mouse VEGFR-2 (Figure 3A). The amino acids surrounding R817 are relatively enriched in the proline residues (Figure 3A) and PRMT4 prefers substrates harboring proline-rich motifs (Shishkova et al., 2017). To demonstrate methylation of R817 on VEGFR-2, we subjected HEK-293 cells co-expressing VEGFR-2 with PRMT4 or R817 mutant VEGFR-2 (A817/VEGFR-2) with PRMT4 and subjected these cells to an *in vivo* methylation assay via ^3^H-S-adenosyl methionine (^3^H-SAM) labeling. The result showed that in the context of overexpression PRMT4, VEGFR-2 is methylated *in vivo*, and mutation of R817 significantly inhibited PRMT4-dependent methylation of VEGFR-2 (Figure 3B).

Methylation of non-histone proteins such as EGFR (Hsu et al., 2011), AKT (Yin et al., 2021), and VEGFR-2 (Hartsough et al., 2013) often modulates phosphorylation of nearby tyrosine and serine/threonine residues. R817 is located two amino acid residues away from Y820 on mouse VEGFR-2 (Y822 on human VEGFR-2) (Figure 3C) suggesting that PRMT4-mediated methylation of R817 on VEGFR-2 could influence phosphorylation of Y820. To address the role of R817 methylation on the phosphorylation of Y820, we developed a rabbit phospho-Y820-specific antibody. Western blot analysis of whole cell lysates from HEK-293 cells expressing wild-type VEGFR-2 or A820/VEGFR-2 showed that Y820 is phosphorylated on VEGFR-2 in a ligand-dependent manner (Figure 3D). Mutation of Y820 did not alter the phosphorylation levels of Y1054 (Figure 3D). Moreover, we show that Y820 is phosphorylated on the endogenously expressed VEGFR-2 in HUVEC-TERT cells (Figure 3E).

### Methylation of R817 modulates phosphorylation of Y820, the interaction of VEGFR-2 with c-Src kinase, and its activation

**Figure 4:**
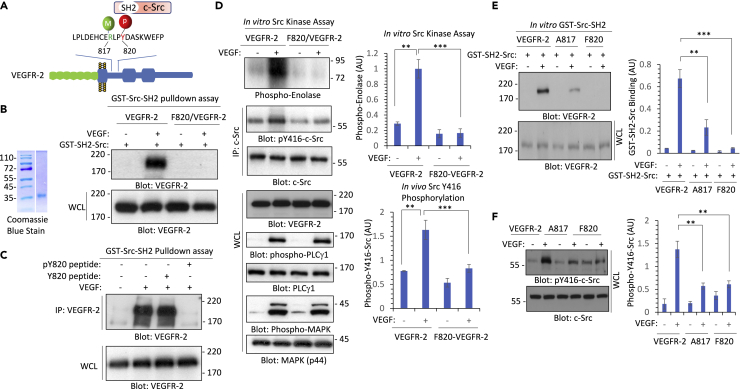
Methylation of arginine 817 facilitates the tyrosine 820-dependent binding of c-Src kinase with VEGFR-2 (A) Schematic of the proposed binding of c-Src with VEGFR-2 and the role of methylation of arginine 817 (R817). Methylation of R817 increases phosphorylation of Y820 leading to the binding of c-Src with VEGFR-2. (B) Coomassie blue staining of GST-Src-SH2. Serum-starved HEK-293 cells expressing wild-type VEGFR-2 or tyrosine 820 mutant VEGFR-2 (F820/VEGFR-2) were stimulated with VEGF for 10 min, cells were lysed, and whole cell lysates were subjected to a GST-Src-SH2 pulldown assay, followed by Western blot analysis using anti-VEGFR-2 antibody. Whole cell lysates (WCL) from the cell groups were also blotted for total VEGFR-2. (C) Serum-starved HEK-293 cells expressing wild-type VEGFR-2 were left unstimulated (−) or stimulated with VEGF (+) for 10 min, whole cell lysates were subjected to GST-Src-SH2 pulldown assay where the GST-Src-SH2 Glutathione Sepharose beads were preincubated with Y820 unphosphorylated peptide or with Y820 phosphorylated peptide, followed by Western blot analysis. Whole cell lysates (WCL) from the cell groups were also blotted for total VEGFR-2. (D) Serum-starved HEK-293 cells expressing wild-type VEGFR-2 and tyrosine mutant 820 VEGFR-2 (F820/VEGFR-2 were left unstimulated (−) or stimulated with VEGF (+) for 10 min, whole cell lysates were immunoprecipitated with anti-Src antibody. The immunoprecipitated samples were divided into two groups. One group was subjected to *in vitro* Src kinase assay using enolase as Src kinase substrate. The remaining immunoprecipitated samples were subjected to Western blot analysis, which were blotted for phospho-Src (pY416) and total Src. Whole cell lysates from the same experimental groups were blotted for VEGFR-2, phospho-PLCγ1, total PLCγ1, phospho-MAPK, and total MAPK. The graphs are representative of three independent experiments. (E) Serum-starved HEK-293 cells expressing wild-type VEGFR-2, F820/VEGFR-2, and A817/VEGFR-2 were left unstimulated (−) or stimulated with VEGF (+) for 10 min, whole cell lysates were subjected to GST-Src-SH2 pulldown assay, followed by Western blot analysis using anti-VEGFR-2 antibody. Whole cell lysates from the same experimental groups were blotted for VEGFR-2. The graph is representative of three independent experiments. (F) Serum-starved HEK-293 cells expressing wild-type VEGFR-2, F820/VEGFR-2, and A817/VEGFR-2 were left unstimulated (−) or stimulated with VEGF (+) for 10 min, whole cell lysates were subjected to Western blot analysis using phospho-Src (pY416) and total Src antibodies. The graph is representative of three independent experiments. ∗∗p-value less than 0.01; ∗∗∗p-value less than 0.001.

To test whether methylation of R817 affects phosphorylation of Y820 on VEGFR-2, we analyzed phosphorylation levels of Y820 in the context of R817 mutant VEGFR-2 (A817/VEGFR-2). Western blot analysis of the whole cell lysates of VEGFR-2/HEK-293 and A817/VEGFR-2/HEK-293 showed that phosphorylation levels of Y820 on the A817/VEGFR-2 were significantly reduced compared to the phosphorylation of Y820 on the wild-type VEGFR-2 (Figure 3F). Next, we investigated whether overexpression of PRMT4 could increase phosphorylation of Y820. PRMT4 increased phosphorylation of Y820 in the context of wild-type VEGFR-2 but not when co-expressed with R817/VEGFR-2 (Figure 3G). The data suggest that PRMT4-mediated R817 methylation modulates Y820 phosphorylation on VEGFR-2. Next, we examined the hypothesis that R817 methylation regulates phosphorylation of Y820 and aids the interaction of c-Src kinase interaction via its Src homology domain 2 (SH2) with VEGFR-2 (Figure 4A). To test our hypothesis, we generated a recombinant GST-Src-SH2 (Figure 4B) and tested its ability to interact with VEGFR-2. GST-Src-SH2 pulldown assay showed that GST-Src-SH2 interacts with the wild-type VEGFR-2 but not with the F820 mutant VEGFR-2 in aVEGF-stimulated manner (Figure 4B). Pre-incubation of GST-SH2-Src with a phospho-Y820 peptide inhibited the binding of GST-SH2-Src, indicating that Src interacts with phospho-Y820 in a dependent manner (Figure 4C).

We asked whether mutation of Y820 impairs the kinase activation of c-Src. We showed that c-Src is activated by the wild-type VEGFR-2 but not by the F820/VEGFR-2 as demonstrated by the phosphorylation of enolase (Src kinase substrate) in an *in vitro* Src kinase assay (Figure 4D). Moreover, Western blot analysis showed that c-Src kinase is phosphorylated at Y416 by the wild-type VEGFR-2 but not by the F820 mutant VEGFR-2 (Figure 4D). Phosphorylation of PLCγ1 or MAPK was not affected by the mutant F820 VEGFR-2 (Figure 4D), indicating that phosphorylation of Y820 selectively regulates c-Src recruitment to VEGFR-2 and its activation. Because mutation of R817 also severely reduced phosphorylation of Y820 on VEGFR-2 (Figure 3E), we examined whether mutation of R817 can affect the binding of c-Src with VEGFR-2. The result showed that the binding of GST-Src-SH2 to mutant R817 VEGFR-2 was significantly reduced compared to the wild-type VEGFR-2 (Figure 4F). Consistent with the reduced binding of GST-Src-SH2 to R817 mutant VEGFR-2, *in vivo* phosphorylation of c-Src by the mutant R817 VEGFR-2 was also markedly reduced (Figure 4G). Taken together, our data demonstrate that methylation of R817 modulates phosphorylation of Y820 and facilitates the recruitment of c-Src to VEGFR-2 and its activation.

### Methylation of R817 regulates VEGFR-2-mediated filopodia protrusions in HEK-293 cells

**Figure 5:**
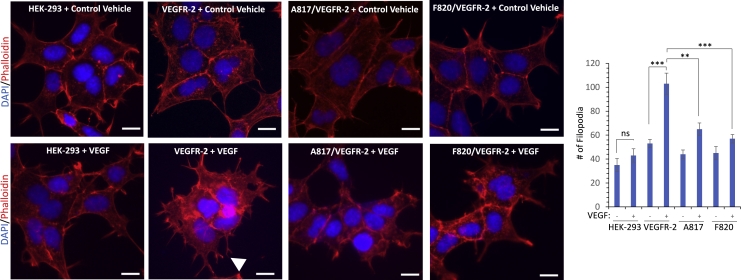
Methylation of arginine 817 and phosphorylation of tyrosine 820 is required for VEGFR-2-induced filopodia protrusions HEK-293 cells expressing wild-type VEGFR-2, F820/VEGFR-2, or A817/VEGFR-2 were either unstimulated or stimulated with VEGF for 30 min. Cells were fixed and stained with phalloidin for actin (red) and DAPI (blue). Three to four pictures were taken from the random fields under the Nikon Deconvolution microscope. Scale bars, 50 μm. Filopodia protrusions wre quantified by the open-source software, filopodyan. White arrow points to filopodia protrusions induced with VEGF-mediated activation of wild-type VEGFR-2. ∗∗p-value less than 0.01; ∗∗∗p-value less than 0.001.

The key angiogenic responses of VEGFR-2 activation such as capillary tube formation, sprouting, and cell migration involve filopodia protrusions of endothelial cells. Similarly, activation of Src family kinases plays a central role in these finely regulated cellular events. Considering the effect of methylation of R817 on the phosphorylation of Y820 and Src activation, we investigated the ability of mutant R817 and Y820 VEGFR-2 constructs expressed in HEK-293 cells in filopodia protrusions. Our analysis showed that stimulation of HEK-293 cells expressing VEGFR-2 with VEGF induced robust filopodia-like structures as detected by phalloidin staining of the cells (Figure 5). However, stimulation of cells expressing mutant R817 with VEGF showed no significant increase in the filopodia-like structures (Figure 5). Similarly, mutant Y820 VEGFR-2 failed to stimulate filopodia formation in HEK-293 cells (Figure 5). Our data indicate that methylation of R817 regulates VEGFR-2-induced filopodia formation.

### Methylation of VEGFR-2 on R817 regulates phosphorylation of Y820 and mediates filopodia in endothelial cells

**Figure 6:**
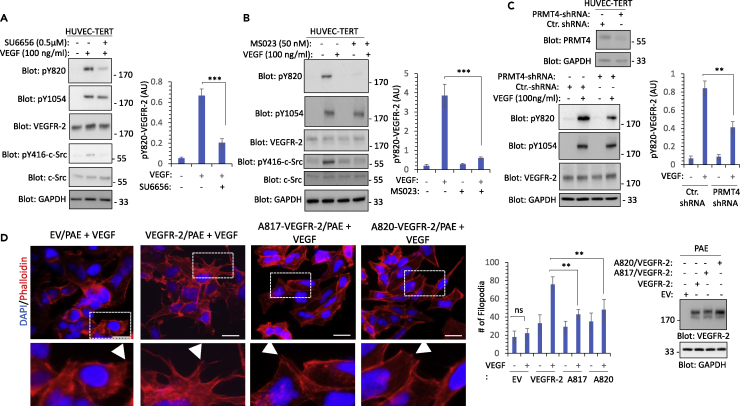
Arginine methylation of VEGFR-2 modulates phosphorylation of Y820 and mediates filopodia in endothelial cells (A) Serum-starved HUVEC-TERT cells were treated with SU6656 for 30 min followed by stimulation of cells with control vehicle (−) or VEGF (100 ng/mL) (+) for 10 min. Whole cell lysates (WCL) were subjected to Western blot analysis using anti-pY820, anti-pY1054, anti-VEGFR-2, anti-pY416-Src, or total c-Src antibody. GAPDH used for protein loading control. The graph is representative of three independent experiments. (B) HUVEC-TERT cells were treated with MS023 for 24 h, followed by stimulation with VEGF for 10 min. Cells were lysed and WCL was subjected to Western blot analysis using antibodies as indicated in the figure legend. The graph is representative of three independent experiments. (C) HUVEC-TERT cells expressing control (ctr) shRNA or PRMT4 shRNA were lysed and WCL was subjected to Western blot analysis using an anti-PRMT4 antibody or anti-GAPDH antibody. Serum-starved HUVEC-TERT cells expressing control (ctr) shRNA or PRMT4 shRNA were left unstimulated (−) or stimulated with VEGF (+) for 10 min. Cells were lysed and WCL was subjected to Western blot analysis using antibodies as indicated. The graph is representative of three independent experiments. (D) PAE cells expressing parental empty vector (EV), wild-type VEGFR-2, A817/VEGFR-2, or AY820/VEGFR-2 were stimulated with VEGF for 30 min. Cells were fixed and stained with phalloidin for actin (red) and DAPI (blue). Three to four pictures were taken from the random fields under the Nikon Deconvolution microscope. Scale bars, 50 μm. Filopodia protrusions quantified as described in Figure 5. ∗∗p-value less than 0.01; ∗∗∗p-value less than 0.001. Not significant (ns). White arrows point to filopodia protrusions. The selected regions (dotted box) are shown in the lower panel. Expression of VEGFR-2 and mutant VEGFR-2 constructs in PAE cells.

VEGFR-2 is predominantly expressed in endothelial cells; therefore, we decided to validate our findings in endothelial cells. Our results showed that treatment of HUVEC-TERT cells with SU6656, a potent Src kinase inhibitor, selectively inhibited phosphorylation of Y820 on VEGFR-2 but not Y1054 (Figure 6A), indicating that phosphorylation of Y820 is mediated by Src kinases in endothelial cells. To investigate the role of methylation of R817 in the phosphorylation of Y820 on the VEGFR-2, we first treated HUVEC-TERT cells with MS023, a selective inhibitor of type I PRMTs, and examined phosphorylation of Y820. Treatment of HUVEC-TERT cells with MS023 inhibited phosphorylation of Y820 but not Y1054, indicating that arginine methylation of VEGFR-2 selectively modulates phosphorylation of Y820 (Figure 6B). Next, we knocked down PRMT4 in HUVEC-TERT cells by shRNA (Figure 6C) and examined phosphorylation of Y820. Depletion of PRMT4 in HUVEC-TERT cells reduced phosphorylation of Y820 by 50% (Figure 6C), indicating that expression of other PRMTs in endothelial cells could also mediate methylation of VEGFR-2 as multiple PRMTs can bind to VEGFR-2 (Figure 1). Mutation of either R817 or Y820 on VEGFR-2 inhibited the ability of VEGFR-2 to promote filopodia formation in HEK-293 cells (Figure 5); thus, we decided to test the role of R817 methylation and phosphorylation of Y820 in filopodia development in the context of endothelial cells. HUVEC-TERT cells endogenously express VEGFR-2 and are not an ideal system to examine the specific functions of R817 or Y820 in the VEGFR-2-mediated cellular events. Therefore, we chose to use porcine aortic endothelial (PAE) cells which do not express VEGFR-2 endogenously; however, upon ectopic expression of VEGFR-2, they respond to the angiogenic responses of VEGFR-2 (Hartsough et al., 2013; Meyer et al., 2011; Rahimi et al., 2000; Singh et al., 2007). Our results showed that stimulation of PAE cells expressing VEGFR-2 with VEGF promoted filopodia protrusions (Figure 6D). However, VEGF stimulation of PAE cells expressing mutant R817 or Y820 VEGFR-2 promoted only a minor filopodia in PAE cells (Figure 6D). Taken together, our results demonstrate that R817 methylation through modulation of phosphorylation of Y820 promotes VEGFR-2-mediated filopodia protrusions in endothelial cells.

## Discussion

In this study, we provide evidence that R817, a proximal residue to Y820 on VEGFR-2, is methylated and this methylation modulates phosphorylation of Y820. PRMT4 through its N-terminal domain interacts with VEGFR-2 and mediates methylation of R817. Methylation of R817 enhances phosphorylation of Y820 and facilitates the recruitment of c-Src to VEGFR-2, leading to increased Src kinase activation and VEGFR-2-induced filopodia formation. Our previous study demonstrated that methylation of a conserved lysine 1041 (K1041) on the VEGFR-2 plays an essential role in the kinase activation of VEGFR-2 by regulating the key autophosphorylation tyrosine 1052 (Y1052). Demonstration that methylation of R817 selectively modulates Y820 phosphorylation and Src recruitment to VEGFR-2 indicates significant crosstalk between the arginine and lysine methylation and tyrosine phosphorylation of VEGFR-2. Interestingly, all the PRMTs (eight PRMTs) tested for their ability to interact with VEGFR-2 in HEK-293 cells, showed a strong binding. However, under more stringent conditions, only PRMT4 strongly interacted with VEGFR-2. One of the distinct features of PRMT4 is the presence of EVH1 and PH domain-like N-terminal domain. We demonstrate that this unique N-terminal domain of PRMT4 specifically interacts with and mediates the binding of PRMT4 with VEGFR-2.

Another important aspect of our study is the demonstration of phosphorylation of Y820 and its role in the recruitment of c-Src kinase to VEGFR-2. Although previous studies have shown that VEGFR-2 mediates c-Src activation (Chou et al., 2002; Eliceiri et al., 1999; Meyer et al., 2002; Sun et al., 2012), however, the precise binding of c-Src with VEGFR-2 has not been fully investigated. Moreover, previous studies have identified multiple tyrosine phosphorylation sites on VEGFR-2. But phosphorylation of Y820 and its functional role in the VEGFR-2 signaling has not been investigated. Our study demonstrates that VEGFR-2 is phosphorylated on Y820 both in the ectopically expressed HEK-293 cells and in the human endothelial cells, HUVEC-TERT, indicating that phosphorylation of Y820 could play an important role in the angiogenic signaling of VEGFR-2. Consistent with this idea, preventing phosphorylation of Y820 via either mutation of Y820, R817, or via pharmacological inhibitors both inhibited c-Src activation and the ability of VEGFR-2 to stimulate filopodia formation in HEK-293 cells and PAE cells. Formation of filopodia protrusions is a key step for many angiogenic responses of endothelial cells such as sprouting and cell migration (Fischer et al., 2019; Zakirov et al., 2021). In agreement with our observation, a recent study demonstrated that mice selectively deficient for c-Src in endothelial displayed significantly reduced blood vessel sprouting and loss in actin-rich filopodial protrusions (Schimmel et al., 2020).

Emerging evidence indicates that PRMTs play broad functional roles in the regulation of endothelial cell function and angiogenesis. For example, endothelial-specific loss of PRMT1 in mice resulted in angiodysplasia (Ishimaru et al., 2017), and PRMT4 has been shown to positively regulate angiogenesis by upregulating VEGF expression (Yan et al., 2021). Similarly, the expression of PRMT5 is required for vasculogenesis in zebrafish (Quillien et al., 2021). Consistent with these observations, targeting PRMTs via pharmacological inhibition has been shown to block angiogenesis (Balcerczyk et al., 2016). Taken together, our study demonstrates a previously unknown mechanism for the role of PRMTs in the regulation of VEGFR-2 signaling and angiogenesis with implications for the development of novel-targeted VEGFR-2 inhibitors.

### Limitations of the study

Our study presented here demonstrates that R817 methylation modulates phosphorylation of Y820, which leads to the recruitment of c-Src kinase and its activation by VEGFR-2. VEGFR-2/Src pathway derives the formation of filopodia protrusions in endothelial cells, which is important for the coordinated angiogenic processes such as sprouting and cellular migration. However, considering that multiple PRMTs can interact with VEGFR-2, it is likely that other PRMTs can mediate the methylation of R817. The role of other PRMTs in the methylation of R817 other than PRMT4 is not analyzed in this study. Moreover, the potential mechanisms by which methylation of R817 modulates phosphorylation of Y820 require further investigation. Methylation of R817 could regulate phosphorylation of Y820 either by altering the activity of a particular tyrosine phosphatase (PTPase) or a tyrosine kinase toward dephosphorylation or phosphorylation of Y820, respectively, but we have not examined these possibilities. Moreover, the direct role of R817 methylation in the VEGFR-2-mediated angiogenesis such as tumor angiogenesis requires further investigation.

## STAR★Methods

### Key resources table

**Table:** 

REAGENT or RESOURCE	SOURCE	IDENTIFIER
**Antibodies**		

pY1054-VEGFR-2	Millipore	Polyclonal rabbit antibody (cat # 07-722)
pY820-VEGFR-2	This paper	N/A
VEGFR2	Millipore	Polyclonal rabbit antibody (07-716-I)
c-Myc tag	CST	Monoclonal rabbit anti-myc tag antibody (71D10) cat #2278
c-Src	CST	Monoclonal rabbit Src (36D10) antibody (cat #2109)
pY416-Src	CST	Monoclonal rabbit Phospho-Src Family (Tyr416) (D49G4, cat #6943)
HRP anti-rabbit IgG	SCBT	Goat HRP anti-rabbit IgG (Cat # sc-2054)
HRP anti-mouse IgG	SCBT	Goat HRP anti-mouse IgG (Cat# sc-2055)
Phalloidin	ThermoFisher	Phalloidin-FITC (Cat# F432)

**Experimental models: cell lines**		

HEK-293 cells	ATCC	Cat # CRL-1573
HUVEC-TERT cells	Amraei et al. (2021)	https://doi.org/10.1021/acscentsci.0c01537

Recombinant DNA		

PRMT1	Open Biosystems	Accession # BC002249
PRMT2	Open Biosystems	Accession # BC079112
PRMT3	Open Biosystems	Accession # BC037544
PRMT4/CARM1	Open Biosystems	Accession # BC036974
PRMT5	Open Biosystems	Accession # BC023905
PRMT6	Open Biosystems	Accession # BC022899
PRMT7	Open Biosystems	Accession # BC000146
PRMT8	Open Biosystems	Accession # BC0022458
PRMT9	Open Biosystems	Accession # BC130445
c-Src	Meyer et al. (2002)	https://www.jbc.org/article/S0021-9258(18)60052-3/fulltext
GST-SH2-Src	Meyer et al. (2002)	https://www.jbc.org/article/S0021-9258(18)60052-3/fulltext
VEGFR-2	Rahimi et al., (2000)	https://www.sciencedirect.com/science/article/pii/S0021925819803022

Oligonucleotides		

PRMT4/CRAM1	SCBT	Human PRMT4/CRAM1 shRNA (Cat # sc-44875-SH)

**Softwareand** algorithms		

ImageJ (FIJI) v. 2.0.0	Schindelin et al., 2012	https://imagej.net/Fiji
Fibriltool	Boudaoud et al. (2014)	https://www.nature.com/articles/nprot.2014.024

### Resource availability

#### Lead contact

Further information and requests for resources and reagents should be directed to and will be fulfilled by the lead contact, Nader Rahimi (nrahimi@bu.edu).

#### Materials availability

All data generated or analyzed during this study are included in this article.

### Experimental model and subject details

#### Cell lines

Human embryonic kidney cells (HEK-293) were purchased from ATCC (Cat# CRL-1573), porcine aortic endothelial cells (PAE) and TERT transformed human umbilical vein endothelial cells (HUVEC-TERT) were maintained in Dulbecco’s Modified Eagle Medium (DMEM) supplemented with 10% fetal bovine serum (FBS), L-glutamine (2 mM), penicillin (50 units/mL) and streptomycin (50 mg/mL). PAE and HUVEC-TERT cells are previously described (Amraei et al., 2021; Rahimi et al., 2000). Other key procedures are described under the method details, below.

### Method details

#### Development of phosphotyrosine 820 antibody

Purified phospho-peptide corresponding to tyrosine 820 to human VEGFR-2 (DELPLDEHCERLPpY_820_DASKWEF) was used to develop polyclonal rabbit anti-phospho-Y820 antibody. The pY820 antibody was affinity purified via Protein-A Sepharose. The specificity of the antibody was further validated using HEK-293 cells expressing VEGFR-2 or mutant Y820-VEGFR-2. The anti-pY820 antibody did not react with the mutant Y820-VEGFR-2, indicating that it specifically recognizes phosphorylated Y820.

#### Cell transfection

HEK-293 cells stably expressing VEGFR-2 were transfected with PRMT constructs via PEI (polyethylenimine) as described (Sun et al., 2021). After 48 h transfection, cells were lysed and subjected to immunoprecipitation or Western blotting as described in the figure legends.

#### Site-directed mutagenesis and viral production

Methylation mutant VEGFR-2 constructs were created using a polymerase chain reaction–based site-directed mutagenesis strategy (Singh et al., 2007). Mutations were confirmed by sequencing. Complementary DNAs were cloned in the Not I and Sal I restriction sites of the retroviral vector pLNCX^2^. Viral production was achieved by transfection into 293-GPG cells, and viral supernatants were collected for 5 days; viral medium was then used as described (Rahimi et al., 2000).

#### Immunoprecipitation and western blot analysis

Cells were grown until 80 to 90% confluence. Cells were lysed with EB lysis buffer (10 mM Tris-HCl, 10% glycerol, pH 7.4, 5 mM EDTA, 50 mM NaCl, 50 mM NaF, 1% Triton X-100, 1 mM phenylmethylsulfonyl fluoride, 2 mM Na3VO4, and 20 μg/mL aprotinin), and normalized whole-cell lysates were subjected to immunoprecipitation by incubation with appropriate antibodies as shown in the figure legends. Immuno-complexes were captured by incubation with either protein A-Sepharose or protein G Agarose beads. The samples were boiled for 5 min at 95°C, the immunoprecipitated proteins were subjected to western blot analysis. In some cases, membranes were stripped by incubation in a stripping buffer (6.25 mM Tris–HCl, pH 6.8, 2% SDS, and 100 mM β-mercaptoethanol) at 50°C for 30 min, washed in Western Rinse buffer (20 mM Tris and 150 mM NaCl), and re-probed with the antibody of interest. The blots were scanned and subsequently quantified using ImageJ (NIH).

#### *In vitro* Src kinase assay

The Src kinase assay was performed as described previously. Briefly, lysates from HEK-293 cells expressing wild type VEGFR-2 or mutant VEGFR-2 constructs were prepared, and equal protein amounts from each cell lysate were immunoprecipitated with anti-Src antibody (Santa Cruz Biotechnology) and half of each immunoprecipitate was assayed for Src kinase activity by incubating with 10 μL of reaction buffer (20 mM PIPES, pH 7.0, 10 mM MnCl2, 10 μmNa3VO4), 2 μg of freshly prepared acid-denatured enolase (Sigma) min, and 10 μCi of [γ-^32^P]. After 30 min of incubation at 30°C, reactions were terminated by the addition of 2× SDS sample buffer, and samples were subjected to SDS-PAGE. The SDS-PAGE gel was fixed in 45% methanol and 10% acetic acid for 30 min at room temperature and dried at 80°C under a vacuum and autoradiographed.

#### *In vitro* methylation assay

*In vitro* methylation assay was performed as described with minor modification (Hartsough et al., 2013). Briefly, VEGFR-2 was immunoprecipitated from HEK-293 cells expressing VEGFR-2 alone or co-expressing VEGFR-2 and PRMT4 were incubated with the methylation reaction buffer ((50 mM Tris pH 8.5, 20 mM KCl, 10 mM MgCl2, 1 mM β-mercaptoethanol, and 100 mM sucrose) plus 1 μL of adenosyl-L-methionine, S-[methyl-3H] (1 mCi/mL stock solution, Perkin Elmer) at 30°C. In some experiments, a purified GST-PRMT4 also added into the reaction mix. The reaction stopped by adding 2× SDS loading buffer after 1 h and was resolved on SDS-PAGE. The separated protein samples were then transferred from the gel to a polyvinylidene difluoride (PVDF) membrane, sprayed with EN3HANCE (Perkin Elmer) and exposed to X-ray film.

#### Purification of GST-PRMT proteins

Recombinant PRMT proteins were purified from the BL21(DE3) *Escherichia coli* transformed with PRMT-pGEX-4T2 constructs. Single colony was grown in 5 mL Luria-Bertani (LB) medium overnight at 37°C. The culture was then expanded into 300 mL LB medium until an optical density of 0.4–0.6. The protein expression was induced by 0.1 mM isopropyl-β-D-thiogalactoside (IPTG) at 30°C for 4–15h. The cells were collected and re-suspended in GST buffer (25 mM Tris pH 8.0, 5 mM dithiothreitol (DTT), 150 mM NaCl) and sonicated (4 cycles/5 s each). After centrifugation, the supernatant was incubated with glutathione Sepharose beads for 2 h at 4°C and subsequently washed four times before use.

#### GST pulldown assay

*In vitro* GST fusion PRMTs or Src-SH2 binding experiments were performed as described previously (Meyer et al., 2008). Briefly, equal numbers of cells expressing VEGFR-2 or mutant VEGFR-2, as described in the figure legends were grown to 90% confluence. Cells were lysed in ice-cold lysis buffer supplemented with 2 mM Na3VO4 and a protease inhibitor cocktail. Equal amounts of the appropriate immobilized GST fusion proteins were incubated with normalized whole-cell lysates by rocking for 3–4 h at 4°C. The beads were washed in the presence of protease inhibitors, and proteins were eluted via boiling at 95°C for 5 min and analyzed by Western blotting using the appropriate antibodies, as described in the figure legends.

#### Immunofluorescence microscopy

HEK-293 cells or PAE cells expressing VEGFR-2 or mutant VEGFR-2 constructs were seeded onto coverslips and grown overnight in 60-mm plates to 70–90% confluence. Cells were serum starved overnight and then either left unstimulated or stimulated with VEGF (100 ng/mL) for 30 min. Cells were washed once with PBS and immediately fixed with freshly prepared 4% paraformaldehyde for 15 min at room temperature. After being washed three more times with PBS, the cells were permeabilized with 0.25% Triton X-100 in Western rinse buffer for 10 min at room temperature and then washed three times with PBS. Cells were stained with Phalloidin-FITC and DAPI and the slides were viewed under the Nikon Deconvolution microscope equipped with a camera and 3-4 pictures were taken per group. Quantification of filopodia was carried out using open-source software, filopodyan as previously described (Rahimi et al., 2021).

### Quantification and statistical analysis

Experimental data were subjected to a Student’s t test or one-way ANOVA, where appropriate, with a representation of at least three independent expderiments. p < 0.05 was considered significant, or as indicated in the figure legends.

## Data Availability

No data is used in this study. Accession numbers for the cDNAs used in this study are listed in the key resources table and the plasmids are available from the lead contact upon request. Code availability. This paper does not report original code. Any additional information required for the data reported in this paper is available from the lead contact upon request.
